# Automatic identification of pavement cracks in public roads using an optimized deep convolutional neural network model

**DOI:** 10.1098/rsta.2022.0169

**Published:** 2023-09-04

**Authors:** Zhihan Lv, Chen Cheng, Haibin Lv

**Affiliations:** ^1^ Department of Game design, Faculty of Arts, 752 36 Uppsala, Uppsala University, Sweden; ^2^ The Second Monitoring and Application Center, CEA, Xìan, People's Republic of China; ^3^ North China Sea Offshore Engineering Survey Institute, Ministry Of Natural Resources North Sea Bureau, People's Republic of China

**Keywords:** pavement distress, mask region-based convolutional neural network, transverse crack, longitudinal crack, mesh crack

## Abstract

The current study aims to improve the efficiency of automatic identification of pavement distress and improve the status quo of difficult identification and detection of pavement distress. First, the identification method of pavement distress and the types of pavement distress are analysed. Then, the design concept of deep learning in pavement distress recognition is described. Finally, the mask region-based convolutional neural network (Mask R-CNN) model is designed and applied in the recognition of road crack distress. The results show that in the evaluation of the model's comprehensive recognition performance, the highest accuracy is 99%, and the lowest accuracy is 95% after the test and evaluation of the designed model in different datasets. In the evaluation of different crack identification and detection methods, the highest accuracy of transverse crack detection is 98% and the lowest accuracy is 95%. In longitudinal crack detection, the highest accuracy is 98% and the lowest accuracy is 92%. In mesh crack detection, the highest accuracy is 98% and the lowest accuracy is 92%. This work not only provides an in-depth reference for the application of deep CNNs in pavement distress recognition but also promotes the improvement of road traffic conditions, thus contributing to the progression of smart cities in the future.

This article is part of the theme issue 'Artificial intelligence in failure analysis of transportation infrastructure and materials'.

## 1. Introduction

Since the twentieth century, especially after the Second World War, cars have become a common means of transportation for the masses, and traffic accidents have gradually become the most common personal injury accidents. At the same time, the road has suffered great damage due to long-term traffic operation, so the distress on the road surface is becoming increasingly complicated with the service time of the road [1]. The emergence of pavement distress recognition technology based on visual technology provides many intelligent recognition methods for pavement distress recognition. With strong advantages in image recognition, deep convolutional neural networks (CNNs) provide a prominent means for pavement distress recognition. Therefore, deep optimization design of pavement distress recognition technology through deep CNN is an innovative study [2].

Hameed *et al*. [3] proposed that the present pavement distress plays an important role in urban development, but the emergence of pavement distress seriously blocks the progression of urban road traffic. Therefore, comprehensive detection of roads is conducted. Identifying and resolving pavement distress is an important task in current society [3]. Han *et al*. [4] noted that pavement distress detection plays an important role in road maintenance [4]. D'Alessandro *et al*. [5] mentioned that pavement distress recognition technology needs to breakthrough manual operation and relies on scientific and technological means. It is innovative to optimize the pavement distress recognition programme by changing the application technology. Khan *et al*. [6] pointed out that to change the traditional methods of pavement distress recognition, image-based pavement distress detection has become a subject of competitive research in various countries. It takes advantage of high-speed and high-precision cameras to shoot road images quickly and uses computers for fast processing to obtain distress information [6]. Lagree *et al*. [7] proposed that in recent years, based on the continuous progression of pavement distress recognition technology, CNNs have attracted extensive attention due to their ability to automatically extract image feature expressions. Some attempts have been made to apply it to crack detection [7]. Zhou [8] pointed out that CNNs and intelligent algorithms can detect the types of pavement distress and automatically identify road conditions, helping road maintenance departments carry out daily maintenance work conveniently, efficiently and in a targeted manner [8].

In summary, first, the recognition technology of pavement distress is analysed. Then, the application of deep learning is discussed. Finally, the mask region-based convolutional neural network (Mask R-CNN) model is designed and applied to pavement distress detection. This work provides technical support for the optimization of pavement distress recognition technology and contributes to the improvement of road construction in future smart cities.

## 2. Pavement distress recognition and deep learning optimization

### (a) Multiple distress identification on public roads

With the continuous development and progress of society, people's daily lives have been inseparable from traffic. Moreover, the speed of road surface damage has accelerated, and the detection of distress on road surfaces has become increasingly heavy [9].

Among various types of urban pavement distress, crack distress is one of the most representative types of pavement distress, which is the early manifestation of other large-scale pavement distress [10]. If not repaired in time, it will develop into a more serious pavement distress type. Compared with other pavement distress types, it is more difficult to detect them [11]. In addition, pavement distress also includes potholes, ruts, looseness, subsidence and surface damage, which have a serious impact on road traffic [12]. Therefore, pavement distress is the main hazard of urban road traffic. Therefore, the accurate identification of cracks and distress on public roads through scientific and technological means plays an important role in the timely handling of distress and reducing traffic losses on public roads [13]. Common road surface distress mainly includes cracks, potholes, ruts, looseness and subsidence. The designed model carries out the comprehensive identification and detection of road pavement cracks, among which the types of cracks mainly include transverse cracks, longitudinal cracks and mesh cracks [14].

### (b) Convolutional neural network-based highway distress recognition

**Figure 1.**
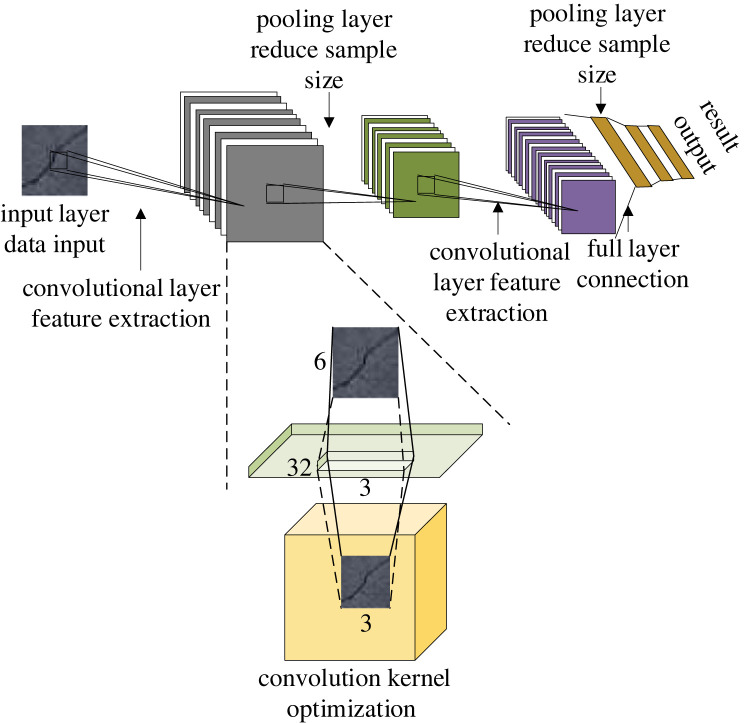
Model of CNN and construction of its convolutional kernel.

Deep learning technology is a prominent technology in machine learning, which has important research significance in the current society. Its application fields are very wide, and its functions are also very complete, which can efficiently address various technical problems in the current society. In the field of deep learning, image recognition and feature extraction are widely studied. Deep CNN performs well in this task [15]. A deep CNN is a multi-layer alternating perceptron that can efficiently detect image features through its multi-layer comprehensive calculation and can extract image features as the technical basis of image research [16]. Figure 1 shows the CNN model and the specific structure principle of the convolutional kernel.

In figure 1, the convolution layer is the main computing layer in the CNN model, and the size and step of its convolution kernel determine its computing mode and computing capability [17]. The commonly used excitation functions are the sigmoid function and ReLU function, and their calculation equations are as follows:

2.1 $$f(x)=\frac{1}{1+e^{-x}},$$

and

**Figure 2.**
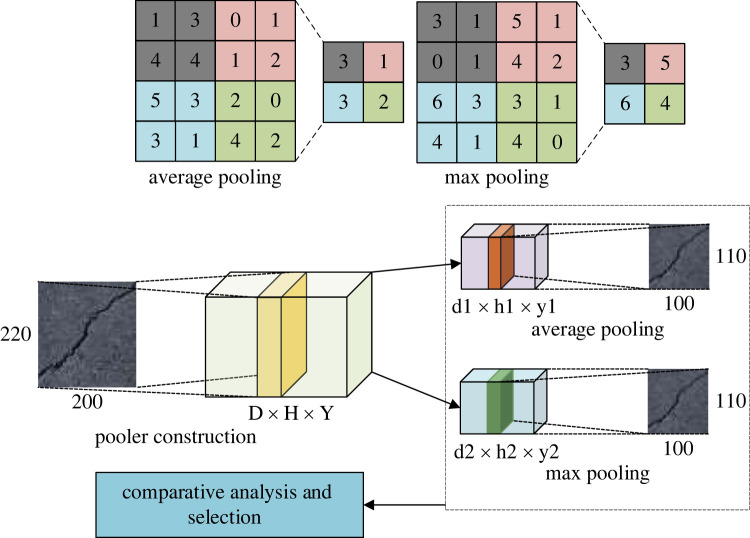
Two pooling operations for the pooling layer and their specific design.

2.2 $$f(x)=\max(0,x)=\left\{ \begin{array}{l} 0,\;\;x<0\\ x,\;\;x\geq 0 \end{array}\right.,$$

where *x* represents the calculation factor. Common pooling operations include average pooling and maximum branch pooling [18]. Figure 2 shows the two pooling operations for the pooling layer and how they work.

In figure 2, the application of the pooling layer greatly optimizes the computational efficiency of the CNN model. The fully connected layer is the terminal structure of a CNN, which is usually used to classify image features. The softmax regression classifier is commonly used, and its expression is

2.3 $$S_{j}=\frac{e^{z_{j}}}{\sum_{k}e^{z_{k}}},$$

where *k* represents the number of neurons, *j* represents neurons and its output can be calculated as follows:

2.4 $$z_{j}=\sum wx+b,$$

where *w* represents the calculated weight, and *b* represents the bias of the neural network layer. The CNN model includes forward operation and reverse operation. Forward operation refers to the process of network training, which takes the calculation results of the former layer as the input of the latter layer to calculate the classification score and the probability of the current class. The reverse operation refers to reversing the output errors of the output layer to the input layer to optimize the model parameters. The error calculation formula is

2.5 $$E(W,b)=\frac{1}{2}\overset{N}{\underset{i=1}{\sum }}∥t_{i}-y_{i}{∥}^{2},$$

where *t* represents the true value of the sample, *i* represents the serial number of the sample, *y* represents the predicted value of the sample and *W* and *b* represent the weight and bias of the neural network layer, respectively. The updating formula of the two is

2.6 $$W^{l}=W^{l}-\eta \frac{\partial }{\partial W^{l}}E(W,b),$$

and

2.7 $$b^{l}=b^{l}-\eta \frac{\partial }{\partial b^{l}}E(W,b),$$

where *l* represents the level of the neural network and *η* represents the learning rate of the model. Typical CNN models include the visual geometry group net model, which uses 3 × 3 convolutional kernels to form a deep network.

## 3. Design of the mask region-based convolutional neural network-based highway distress recognition model

**Figure 3.**
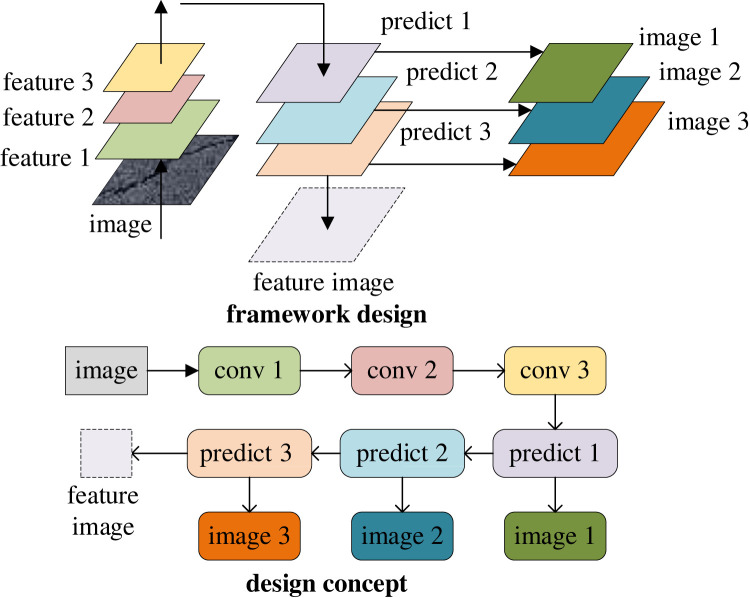
Optimal design of FPN.

To optimize the model, a Mask R-CNN model is designed to detect and recognize pavement distress. In the task of pavement crack identification, the detection network can be used to locate the crack, but it cannot calculate the crack length, width, area and other parameters [19]. This model using a single task (such as a detection network or segmentation network) can only complete the detection or segmentation task of road distress [20]. Moreover, the advantages of the Mask R-CNN model can be highlighted by the feature pyramid network (FPN), as illustrated in figure 3, which shows the optimization design of the model convolution process by FPN.

**Figure 4.**
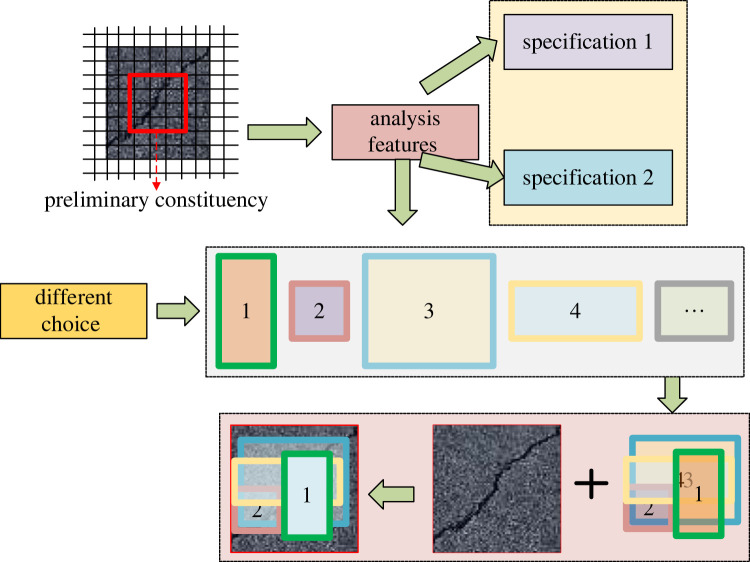
Optimal design of the RPN generation candidate box.

Figure 3 also shows the optimization design of the convolution process for the FPN network structure. Since the original Mask R-CNN algorithm uses the ResNet residual network as the skeleton network, which has good performance, is easy to train, and can be stacked with many layers, ResNet is used as the basic network, and the FPN idea is added for illustration [21]. Its calculation is as follows:

3.1 $$k=k_{0}+\text{lo}{\text{g}}_{2}\left(\sqrt{\text{wh}}/224\right),$$

where *k* represents the feature graph, and *w* and *h* represent the width and height of the feature graph, respectively. Then, the model's job is to generate the RPN of the candidate region, which is to generate a high-quality region candidate box. Figure 4 shows the optimized design of the RPN generation candidate box for model work.

In figure 4, the red box on the left represents a convolution kernel, and a new feature graph is generated after sliding calculation. In addition, boundary regression is used to make the positions of the candidate boxes more accurate. Usually, (*x, y, w, h*) is used to represent the window of candidate boxes [22]. Then, the calculation of boundary regression is as follows:

3.2 $$f(P_{x},P_{y},P_{w},P_{h})=({\hat{G}}_{x},{\hat{G}}_{y},{\hat{G}}_{w},{\hat{G}}_{h})\approx (G_{x},G_{y},G_{w},G_{h}),$$

where *f* represents the mapping, *P* represents the candidate box of prediction, *G* represents the box of real position and $\hat{G}$ represents the box of regression window. The principle of the equation is moving the prediction candidate box to the regression window box close to the real position through mapping. The specific calculation is as follows:

3.3 $$\begin{array}{rl} \Delta x & \;=P_{w}d_{x}(P), \end{array}$$

3.4 $$\begin{array}{rl} \Delta y & \;=P_{h}d_{y}(P), \end{array}$$

3.5 $$\begin{array}{rl} {\hat{G}}_{x} & \;=P_{w}d_{x}(P)+P_{x}, \end{array}$$

3.6 $$\begin{array}{rl} {\hat{G}}_{y} & \;=P_{h}d_{y}(P)+P_{y}, \end{array}$$

3.7 $$\begin{array}{rl} S_{w} & \;=\text{exp}(d_{w}(P)), \end{array}$$

3.8 $$\begin{array}{rl} S_{h} & \;=\text{exp}(d_{h}(P)), \end{array}$$

3.9 $$\begin{array}{rl} {\hat{G}}_{w} & \;=P_{w}\text{exp}(d_{w}(P)) \end{array}$$

3.10 $$\begin{array}{rl} \text{and}\;{\hat{G}}_{h} & \;=P_{h}\text{exp}(d_{h}(P)), \end{array}$$

where Δ represents position shift and *S* represents scale scaling. Then, the calculation of translation and scale scaling is as follows:

3.11 $$\begin{array}{rl} t_{x} & \;=\frac{\left(G_{x}-P_{x}\right)}{P_{w}}, \end{array}$$

3.12 $$\begin{array}{rl} t_{y} & \;=\frac{\left(G_{y}-P_{y}\right)}{P_{h}}, \end{array}$$

3.13 $$\begin{array}{rl} t_{w} & \;=\text{log}\left(\frac{G_{w}}{P_{w}}\right), \end{array}$$

3.14 $$\begin{array}{rl} t_{h} & \;=\text{log}\left(\frac{G_{h}}{P_{h}}\right) \end{array}$$

**Figure 5.**
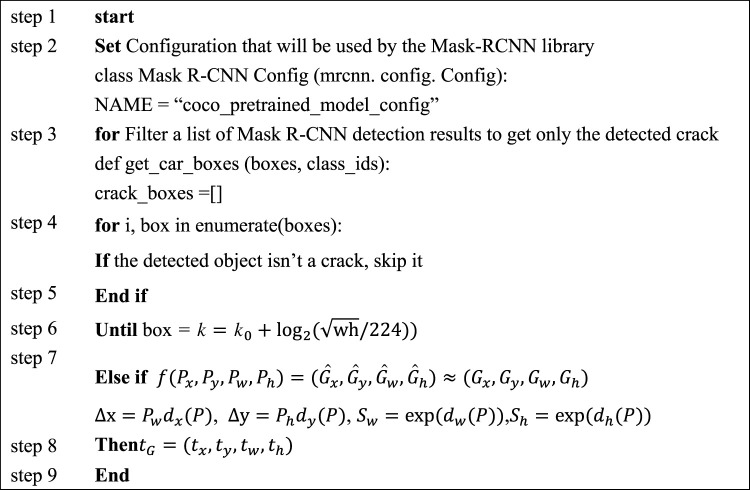
Specific calculation flow of the Mask R-CNN model.

3.15 $$\begin{array}{rl} \text{and}\;t_{G} & \;=(t_{x},t_{y},t_{w},t_{h}), \end{array}$$

where *t_G_* represents the real coordinates. Figure 5 shows the specific calculation flow of the model.

In figure 5, the model predicts pavement distress through calculation and then detects and evaluates pavement distress [23]. Then, the loss function of the model can be calculated by

3.16 $$\text{Loss}=\overset{N}{\underset{i}{\sum }}{\left(t_{*}^{i}-\hat{w_{*}^{T}}\emptyset_{5}(P^{i})\right)}^{2}$$

and

3.17 $$W_{*}=\text{arg}\underset{w*}{\text{min}}\overset{N}{\underset{i}{\sum }}{\left(t_{*}^{i}-\hat{w_{*}^{T}}\emptyset_{5}(P^{i})\right)}^{2}+\lambda ∥{\hat{w}}_{*}{∥}^{2},$$

where $\emptyset_{5}$ represents the feature vector for the candidate area, $W_{*}$ represents the learning parameter and $\hat{w_{*}^{T}}\emptyset_{5}$ represents the prediction result [24]. The regression functions of the model are as follows:

3.18 $$\begin{array}{rl} t_{x} & \;=\frac{\left(x-x_{a}\right)}{w_{a}}, \end{array}$$

3.19 $$\begin{array}{rl} t_{y} & \;=\frac{\left(y-y_{a}\right)}{h_{a}}, \end{array}$$

3.20 $$\begin{array}{rl} t_{w} & \;=\text{log}\left(\frac{w}{w_{a}}\right), \end{array}$$

3.21 $$\begin{array}{rl} t_{h} & \;=\text{log}\left(\frac{h}{h_{a}}\right), \end{array}$$

3.22 $$\begin{array}{rl} t_{x}^{*} & \;=\frac{\left(x^{*}-x_{a}\right)}{w_{a}}, \end{array}$$

3.23 $$\begin{array}{rl} t_{y}^{*} & \;=\frac{\left(y^{*}-y_{a}\right)}{h_{a}}, \end{array}$$

3.24 $$\begin{array}{rl} t_{w}^{*} & \;=\text{log}\left(\frac{w^{*}}{w_{a}}\right) \end{array}$$

3.25 $$\begin{array}{rl} \text{and}\;t_{h}^{*} & \;=\text{log}\left(\frac{h^{*}}{h_{a}}\right), \end{array}$$

where *a* stands for the anchor point. Finally, the region of interest (ROI) align operation of the model is executed, including linear interpolation and bilinear interpolation. Its task is to accurately infer the remaining relevant data from the given data [24]. The calculation of linear interpolation is as follows:

3.26 $$\begin{array}{rl} y & \;=\omega_{0}y_{0}+\omega_{1}y_{1}, \end{array}$$

3.27 $$\begin{array}{rl} \frac{y-y_{0}}{x-x_{0}} & \;=\frac{y-y_{1}}{x-x_{1}} \end{array}$$

3.28 $$\begin{array}{rl} \text{and}\;y & \;=\frac{x-x_{0}}{x_{1}-x_{0}}y_{1}+\frac{x_{1}-x}{x_{1}-x_{0}}y_{0}, \end{array}$$

where *ω* represents the weight. The calculation of bilinear interpolation is as follows:

3.29 $$\begin{array}{rl} f(R_{1}) & \;=\frac{x_{2}-x}{x_{2}-x_{1}}f(Q_{11})+\frac{x-x_{1}}{x_{2}-x_{1}}f(Q_{21}), \end{array}$$

3.30 $$\begin{array}{rl} f(R_{2}) & \;=\frac{x_{2}-x}{x_{2}-x_{1}}f(Q_{12})+\frac{x-x_{1}}{x_{2}-x_{1}}f(Q_{22}) \end{array}$$

3.31 $$\begin{array}{rl} \text{and}\;f(P) & \;=\frac{y_{2}-y}{y_{2}-y_{1}}f(R_{1})+\frac{y-y_{1}}{y_{2}-y_{1}}f(R_{2}) \end{array}$$

where *R*_1_, *R*_2_ and *P* represent two given points and a calculated point, and *Q*_11_, *Q*_21_ and *Q*_22_ are transition points used in the calculation. Linear and bilinear interpolation operations are the basis of the ROI align operation [23]. The model designed in this work is aimed at the recognition of multiple road diseases, so multi-task loss is designed. The relevant loss functions include the classification loss function, regression loss function and segmentation loss function [25].

The designed model is for the identification of multiple distress on the road, so multi-task loss is designed. Related loss functions include the classified loss function, regression loss function and segmentation loss function [25], and their calculations are as follows:

3.32 $$\begin{array}{rl} L_{\text{cls}} & \;=-\sum_{i=1}^{M}y\text{log}(p), \end{array}$$

3.33 $$\begin{array}{rl} L_{\text{box}} & \;=\sum_{i\in x,y,w,h}smooth(t_{i}-t_{i}^{*}), \end{array}$$

3.34 $$\begin{array}{rl} smoothL_{1}(x) & \;=\left\{ \begin{array}{l} 0.5x^{2},|x|<1\\ |x|-0.5,\text{others} \end{array}\right. \end{array}$$

**Figure 6.**
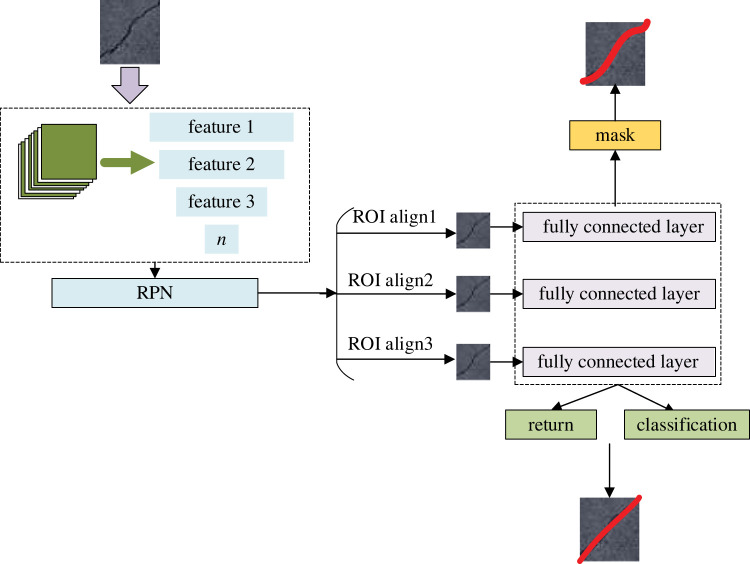
Comprehensive design of the Mask R-CNN model.

3.35 $$\begin{array}{rl} \text{and}\;L_{\text{mask}} & \;=-\frac{1}{a^{2}}\underset{1\leq i,j\leq a}{\sum }[y_{ij}\log{\hat{y}}_{ij}^{n}+(1-y_{ij})\log(1-{\hat{y}}_{ij}^{n})], \end{array}$$

where *i* and *j* represent pixels, *y* represents pixel labels, ${\hat{y}}_{ij}^{n}$ represents predicted values and *n* represents the hierarchy of the network. Other parameters are the same as the above equations. Figure 6 shows the comprehensive design of the optimized Mask R-CNN model.

In figure 6, the Mask R-CNN model is comprehensively optimized, thereby improving the recognition efficiency of the model for road diseases and providing technical support for the improvement of road traffic conditions [26]. The workflow of the model is first to decolour the collected image and then to extract features from the grey image. In this process, the model will also carry out convolution and pooling operations on the graph to effectively extract image features and ensure the working efficiency and final effect of the model.

## 4. Experimental data setting

In this work, the data used for training and evaluation of the design model are from public datasets, including the German asphalt pavement (GAP) 384 dataset [27], which contains a total of 1969 grey value images. The image resolution is 1920 × 1080 pixels, and each pixel represents 1.2 × 1.2 mm. The Cracktree 200 dataset [28] includes 206 road surface images of size 800 × 600 with various types of cracks. The Cracks and Potholes in Road Images dataset [29] is mainly a collection of defective images of paved roads in Brazil. It contains 2235 images from highways, including roads, cracks and potholes. The structural defects network (SDNET) 2018 dataset [30] contains more than 56 000 images of cracked and non-cracked concrete bridge decks, walls and walkways. The dataset includes cracks as narrow as 0.06 mm and as wide as 25 mm. The dataset also includes images with various obstacles, including shadows, surface roughness, scaling, edges, holes and background debris. In the identification of road defects, the model designed in this work includes lateral cracks, longitudinal cracks and mesh cracks. The main method aims to preliminarily identify and detect the length of various cracks through the input test model of the images in the dataset, of which a total of 500 images are detected in this work. In table 1, the basic information of the computer hardware devices for simulation detection in this work is displayed.

**Table 1.** Experimental configuration.

serial number	experiment apparatus	configure
1	operating system	Windows10
2	CPU	Intel(R)_Xeon(R)_W-2133
3	memory	32G
4	graphics card	Nvidia GTX 2080Ti
5	programming language	Python3.9
6	frame type	Pytorch

## 5. Evaluation of convolutional neural network-based pavement distress recognition model

### (a) Comparative evaluation of optimization performance of convolutional neural network model

**Figure 7.**
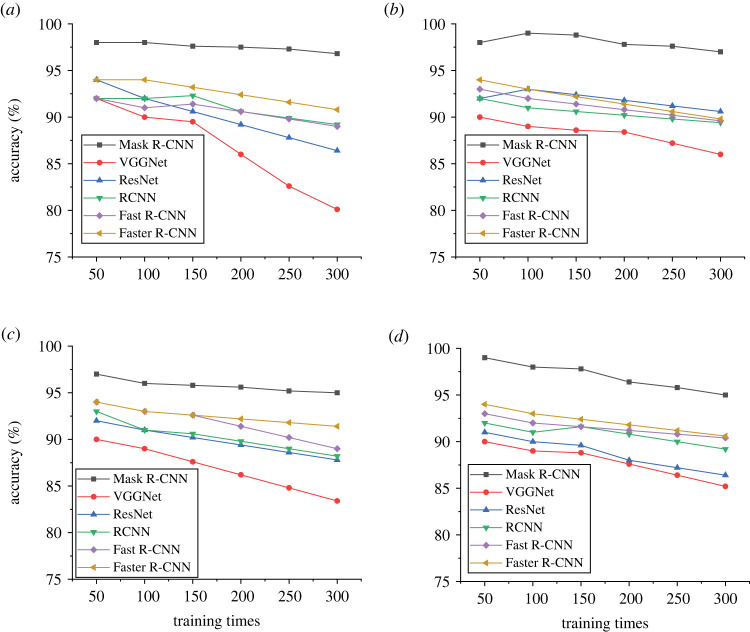
Evaluation of pavement distress detection accuracy ((*a*) the GAP dataset, (*b*) the Cracktree 200 dataset, (*c*) the CPRI dataset and (*d*) the SDNET dataset).

By designing and optimizing the Mask R-CNN model, highway distress recognition technology is realized to comprehensively improve the model's recognition and detection effect and improve the current highway distress detection status. Figure 7 shows the evaluation results of the crack detection accuracy of the model.

**Figure 8.**
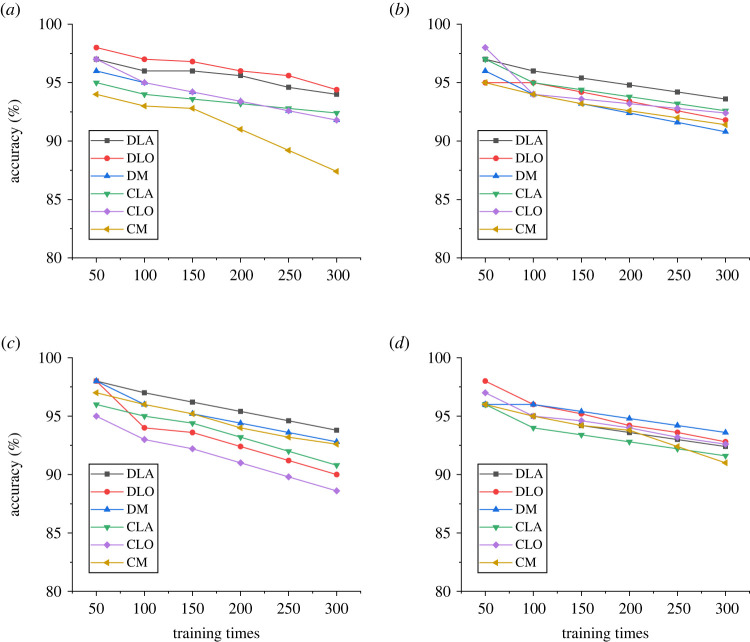
Identification and evaluation of different cracks by the Mask R-CNN model ((*a*) the GAP dataset, (*b*) the Cracktree 200 dataset, (*c*) the CPRI dataset and (*d*) the SDNET dataset).

In the detection of pavement distress cracks, the model is tested and evaluated in four datasets, and it is found that the detection accuracy of the model is 99% at the highest and 95% at the lowest (figure 7). Figure 8 shows the evaluation results of the designed model for the identification of different cracks.

In figure 8, DLA refers to detecting lateral cracks, DLO refers to detecting longitudinal cracks, DM refers to detecting mesh cracks, CLA refers to cutting lateral cracks, CLO refers to cutting longitudinal cracks and CM refers to cutting mesh cracks. The results show that the highest accuracy of the model is 98% and the lowest accuracy is 87% in transverse crack detection.

### (b) Mask region-based convolutional neural network model detection performance evaluation

**Figure 9.**
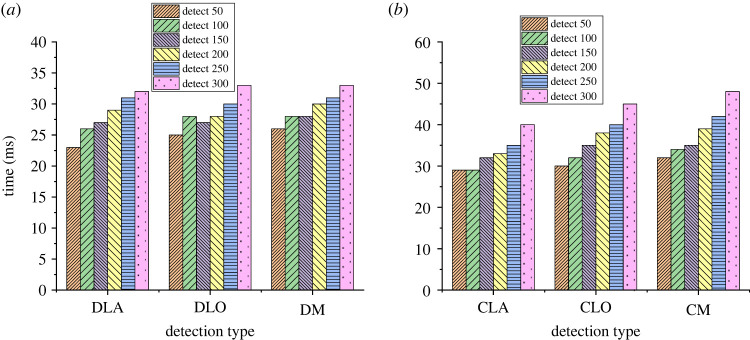
Evaluation of detection time for different crack lengths ((*a*) detection, (*b*) cutting).

The optimized model can calculate the specific length of cracks by predicting the movement distance of the frame. Therefore, this model is a breakthrough of traditional crack identification technology to a certain extent and provides a more advantageous model for the identification of cracks. Figure 9 shows the evaluation results of the test time for different crack lengths.

**Figure 10.**
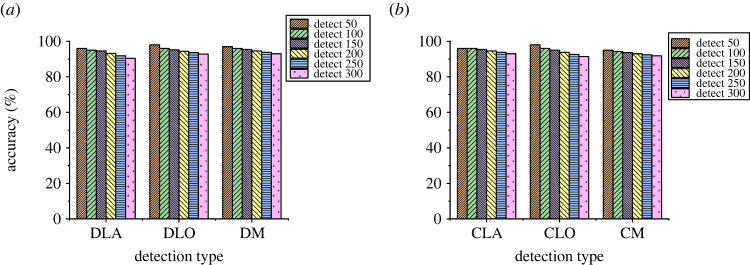
Evaluation of the length detection accuracy of the model ((*a*) detection, (*b*) cutting).

In figure 9, the legend represents the detection times. The results show that the longest detection time of the model is 32 ms, the lowest is 25 ms, the longest cutting time of the model is 48 ms and the lowest is 30 ms. In addition, the length detection and cutting accuracy of the model are very high. Figure 10 shows the evaluation results of the length detection accuracy of the model.

**Figure 11.**
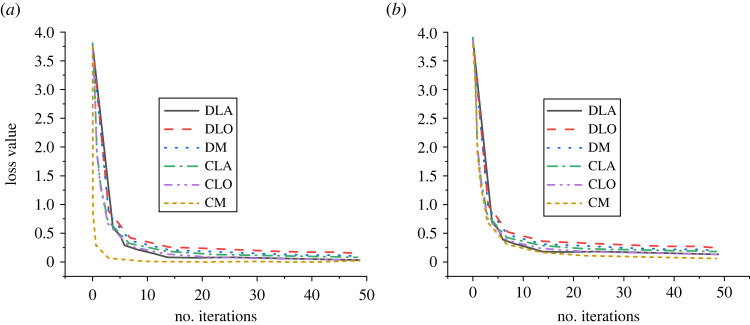
Loss values in pavement distress detection ((*a*) the loss of crack recognition, (*b*) the loss of crack length detection).

In figure 10, in the detection of pavement cracks, the model has a very high detection accuracy of length, with the highest detection accuracy 98% and the lowest detection accuracy 90%. In the cutting process, the accuracy of the model is 98% and 92%, respectively. Figure 11 shows the loss value evaluation results of this model in road surface distress detection.

In figure 11, the loss value of the model in the detection is very low. In crack identification, when the number of loss iterations of the model is 7, the loss value is close to the minimum value and the minimum loss value of the model is maintained at 0.25. In crack length detection, the loss value of the model is slightly higher, but when the number of zone times of the model is 10 times, it is close to the lowest and tends to be stable, while the loss value of the model is 0.5.

## 6. Conclusion

With the development of the economy, road traffic has been affected by many kinds of transportation tools, so the road is inevitably damaged. Based on this, the recognition technology of road surface diseases and the types of road diseases are discussed. Then, the application design of deep learning technology in road disease recognition is introduced. Finally, based on deep CNN technology, the Mask R-CNN model is designed for the recognition of road crack disease, and the designed model is comprehensively evaluated and detected. The results show that in the evaluation of model comprehensive recognition performance, the designed model is tested and evaluated in different datasets, and the detection accuracy of the model is the highest, which is superior to other recognition techniques in all aspects. Second, the accuracy of the designed model is always higher than 92% in transverse fracture detection, longitudinal fracture detection and mesh fracture detection. The accuracy of transverse cracks, longitudinal cracks and mesh cracks is always higher than 87%. In the crack length detection, the longest detection time of the model is approximately 32 ms, and the shortest detection time is approximately 25 ms. The longest cutting time of the model is approximately 48 ms, and the shortest is approximately 30 ms. In the detection accuracy, the highest is approximately 98% and the lowest is approximately 90%. In cutting, the highest accuracy of the model is approximately 98% and the lowest is approximately 92%. Finally, the loss value of this model in detection is very low. In fracture identification, when the number of loss iterations of the model is approximately 7, the loss value is close to the minimum value of approximately 0.25. In fracture length detection, the loss value of the model is slightly higher, but the model is close to the lowest loss value of approximately 0.5 when the number of iterations is approximately 10. Although a better model is designed and comprehensive evaluation results are provided in this work, the application effect of the model in the actual environment is not tested in the model evaluation. At the same time, the evaluation factors used are not comprehensive enough, so more comprehensive reference factors will be designed in future research to further evaluate the performance of the model and design a more comprehensive model application concept.

## Data Availability

The raw data supporting the conclusions of this article are available upon request.
